# Construction of a ceRNA coregulatory network and screening of hub biomarkers for salt‐sensitive hypertension

**DOI:** 10.1111/jcmm.15285

**Published:** 2020-05-15

**Authors:** Ling Zhang, Han Qi, Zheng Liu, Wen‐Juan Peng, Han Cao, Chun‐Yue Guo, Yan‐Yan Sun, Christine Pao, Yu‐Tao Xiang

**Affiliations:** ^1^ Department of Epidemiology and Health Statistics School of Public Health Beijing Municipal Key Laboratory of Clinical Epidemiology Capital Medical University Beijing China; ^2^ The National Clinical Research Center for Mental Disorders Beijing Key Laboratory of Mental Disorders & the Advanced Innovation Center for Human Brain Protection Beijing Anding Hospital School of Mental Health Capital Medical University Beijing China; ^3^ Science Department Peking University People's Hospital Beijing China; ^4^ Department of Psychiatry University of North Carolina at Chapel Hill Chapel Hill NC USA; ^5^ Unit of Psychiatry Institute of Translational Medicine Faculty of Health Sciences University of Macau Macao China

**Keywords:** biomarkers, competing endogenous RNA, long non‐coding RNA, networks, salt‐sensitive hypertension

## Abstract

Salt‐sensitive hypertension (SSH) is an independent risk factor for cardiovascular disease. The regulation of long non‐coding RNAs, mRNAs and competing endogenous RNAs (ceRNAs) in the pathogenesis of SSH is uncertain. An RNA microarray was performed to discover SSH‐associated differentially expressed lncRNAs (DElncRNAs) and mRNAs (DEmRNAs), and 296 DElncRNAs and 44 DEmRNAs were identified, and 247 DElncRNAs and 44 DEmRNAs among these RNAs were included in the coexpression network. The coregulatory network included 23 ceRNA loops, and six hub RNAs (lnc‐ILK‐8:1, lnc‐OTX1‐7:1, lnc‐RCAN1‐6:1, *GIMAP8*, *SUV420H1* and *PIGV*) were identified for further population validation. The ceRNA correlations among lnc‐OTX1‐7:1, hsa‐miR‐361‐5p and *GIMAP8* were confirmed in SSH and SRH patients. A larger‐sample validation confirmed that *GIMAP8, SUV420H1* and *PIGV* were differentially expressed between the SSH and SRH groups. In addition, *SUV420H1* was included in the SSH screening model, and the area under the curve of the model was 0.720 (95% CI: 0.624‐0.816). Our study explored the transcriptome profiles of SSH and constructed a ceRNA network to help elucidate the mechanism of SSH. In addition, *SUV420H1* was identified as a hub element that participates in SSH transcriptional regulation and as a potential biomarker for the early diagnosis of SSH.

## 1 INTRODUCTION

Salt‐sensitive hypertension (SSH) is an intermediate inheritance phenotype of essential hypertension associated with inter‐individual differences and genetic predisposition.^1^ Individuals who exhibit increases in blood pressure due to high‐salt intake are defined as salt‐sensitive (SS), whereas other individuals are salt‐resistant (SR).^2^ The prevalence of SSH is higher in aged individuals, black Americans and females groups,^3^, ^4^, ^5^ and a high prevalence of salt sensitivity is reportedly a feature of hypertension in Asia.^6^ Identification of the salt sensitivity among middle‐ to old‐aged populations in China is important for providing advices on salt reduction and healthy habits. It has been found that SSH is related to increased risks of age‐related hypertension and cardiovascular events.^7^, ^8^ Additionally, SSH rats and patients exhibit obvious target organ damage, particularly in the renal and cardiovascular systems.^9^, ^10^ However, the pathogenesis of SSH is not completely understood and might be related to, for instance, the renin‐angiotensin‐aldosterone system (RAAS), ion and water channels, the endothelial system, the sympathetic nervous system and the natriuretic peptide system.^11^ Therefore, it is necessary to further investigate the mechanism of SSH and discover the core elements involved in the pathways associated with the prevention, early identification and effective therapy of SSH.

Various studies have indicated that genomic variations are associated with SSH.^12^, ^13^, ^14^ Because the transcriptome serves as the bridge between genomics and biological functions, it can also participate in the pathogenesis of SSH. MicroRNAs (miRNAs) have been identified as biomarkers for the diagnosis of SSH,^15^ and this finding provides insights for the use of other non‐coding RNAs as SSH biomarkers. Long non‐coding RNAs (lncRNAs) are endogenous non‐coding RNAs with a length of more than 200 nucleotides that can regulate gene expression at the epigenetic, transcriptional and post‐transcriptional levels.^16^ However, the regulatory role of lncRNAs is not isolated but is associated with a complex of interacting miRNAs and messenger RNAs (mRNAs). An lncRNA can function as a competing endogenous RNA (ceRNA) to absorb available miRNAs and affect the binding of an miRNA to mRNAs through its own miRNA response element.^17^, ^18^ This RNA‐RNA crosstalk has been widely studied in many chronic diseases, such as cancer,^19^ coronary heart disease ^20^ and myocardial infarction.^21^ However, the functions of ceRNA in essential hypertension, as well as SSH, are not well understood. Thus, the ceRNA coregulatory network of SSH is necessary to further explore the mechanism of SSH.

In addition, due to its features of high conservation and active functions, the dysregulation of lncRNAs has been found in and identified as a biomarker for many diseases, particularly cardiovascular disease.^22^ Several in vitro studies have found that lncRNAs are differentially expressed in Dahl salt‐sensitive rats.^23^, ^24^ The lncRNA NPPA antisense can influence the concentration of atrial natriuretic peptide in vivo through regulation of the alternative splicing of *NPPA* and contributes to the regulation of blood pressure.^25^ Thus, lncRNAs might play roles in the pathogenesis of SSH and act as biomarkers for the identification of SSH. Similarly, various mRNAs, such as *STK39*, have been found to be involved in the pathogenesis of cardiovascular disease in hypertensive patients,^26^ and mRNAs have also been associated with epithelial sodium channels ^27^ and renin ^28^ in SSH rats. Due to the lack of evidence regarding the roles of lncRNAs and mRNAs in SSH patients, we further screened the SSH‐associated differentially expressed lncRNAs (DElncRNAs) and mRNAs (DEmRNAs) from the ceRNA network as hub biomarkers of SSH patients.

In summary, this study aimed to construct a ceRNA network of SSH using data obtained using RNA microarray and bioinformatics technologies, validate the coregulatory relationship among RNAs and screen the utility of the differentially expressed RNAs (DERNAs) that serve as hubs in the ceRNA network for the precise diagnosis of SSH. Additionally, functional enrichment and pathway analyses were performed to explore the potential functions of the hub RNAs, and these findings will provide insights on the mechanism of SSH at the transcriptomic level.

## 2 MATERIALS AND METHODS

### 2.1 Participants and sample collection

A total of 112 hypertensive patients (51 SSH and 61 SRH) were selected from the previously established EpiSS (System Epidemiology Study on Salt Sensitivity of Blood pressure) database.^29^ The participants were enrolled via telephone notification based on the following inclusion criteria: (a) diagnosis of stage one essential hypertension; (b) age of 40‐70 years; (c) living in Beijing for more than 5 years; and (d) Han ethnicity. Patients with severe coronary heart disease, heart failure, stroke, peripheral arterial disease, congenital heart disease, acute myocardial infarction, liver and kidney disease or cancer were excluded. The patients were divided into the SSH (case) and SRH groups (control) according to the modified Sullivan's acute oral saline and diuresis shrinkage test (MSAOSL‐DST) results.^30^, ^31^

Demographic data and blood pressure and physical measurements were obtained by trained investigators using standard questionnaires. Blood collection was performed by professional nurses. The blood collected in EDTA tubes was immediately transferred to RNA blood tubes (BioTeke Corporation) for further RNA experiments. This study was approved by the Ethical Committee of Capital Medical University. Prior to initiation of the study, all the participants were informed of the purpose of the study and signed informed consent forms.

### 2.2 Total RNA extraction and lncRNA‐mRNA microarray

A total RNA blood extraction kit (centrifugal type, BioTeke Corporation) was used to extract RNA from whole blood according to the manufacturer's instructions. An Agilent Bioanalyzer 2100 (Agilent Technologies) was then used to determine the concentration and purity of the RNA sample, and 100 ng of RNA was used for 1.5% agarose gel electrophoresis for preliminary quality control. The RNA samples satisfied the following criteria: concentration ≥80 ng/μL, RIN ≥ 7 and A260/280 value between 1.9 and 2.2. The integrity of the agarose gel electrophoresis requires clearly visible 28S/18S bands without obvious degradation.

The Agilent SBC human (4*180 K) ceRNA array v1.0 was employed to detect the expression of lncRNAs and mRNAs. Ten participants were selected for the microarray test according to their SSH status, age, gender and body mass index (BMI). cRNA was amplified and labelled using the Low Input Quick Amp WT Labelling Kit (Agilent Technologies) following the manufacturer's instructions. Labelled cRNA was purified using a RNeasy Mini Kit (QIAGEN, GmBH). Each slide was hybridized with 1.65 μg of Cy3‐labelled cRNA using a Gene Expression Hybridization Kit in a hybridization oven. An Agilent Microarray Scanner was used to scan the slides. The raw data were normalized using the Quantile algorithm with the ‘limma’ packages in R software. The microarray data are available from the Gene Expression Omnibus (GEO) database under the accession number GSE135111.

### 2.3 Screening for DElncRNAs and DEmRNAs

The significance of DElncRNAs and DEmRNAs was identified based on four criteria: (a) the *P* values for differential expression were lower than 0.001; (b) the fold changes between the SSH and SRH groups were higher than 2 or lower than 0.5; (c) the RNA signal values were significantly different from the background noise; and (d) the expression range was higher than the median of the range of all RNAs or the mean expression signal was higher than the median of mean expression levels of all RNAs.^32^

### 2.4 LncRNA‐mRNA coexpression

A Pearson linear correlation analysis was performed to estimate the lncRNA and mRNA coexpression relationships. The Pearson correlation coefficient (PCC) was calculated using the RNA expression levels. The lncRNA‐mRNA pairs with a PCC ≥ 0.95 were selected for further target gene prediction and gene annotation.

### 2.5 Target gene prediction

Two databases were used to predict the RNA target genes. First, we used miRDB (http://www.mirdb.org/custom.html) to predict the target miRNAs of DElncRNAs. Before the prediction, we obtained the whole sequences of DElncRNAs from LNCipedia.org (http://Incipedia.org/db/search) and then inputted the sequences into miRDB. Second, miRmap (https://mirmap.ezlab.org/app/) was used to predict the target mRNAs of differentially expressed miRNAs (DEmiRNAs). The top ten listed mRNAs were selected as the prediction results.

In addition, the following eight SSH‐associated DEmiRNAs were identified in our previous study through miRNA sequencing and larger‐sample validation: hsa‐miR‐423‐5p, hsa‐miR‐15b‐5p, hsa‐miR‐210‐3p, hsa‐miR‐362‐5p, hsa‐miR‐19a‐3p, hsa‐miR‐26b‐3p, hsa‐miR‐362‐5p and hsa‐miR‐382‐5p.^15^ Therefore, we regarded these eight DEmiRNAs as core elements and selected the DElncRNAs and DEmRNAs that were predicted to be related to these DEmiRNAs.

### 2.6 Construction of ceRNA network

We intersected the mRNAs obtained through target gene prediction and the coexpression analysis to obtain the target DEmRNAs. We then integrated the DElncRNAs, target DEmRNAs and DEmiRNAs to construct an SSH ceRNA network. The edges and nodes in the network represent the interaction relationships and RNAs, respectively. The interactions were based on the ceRNA theory that lncRNAs can act as miRNA sponges to suppress miRNAs and regulate the expression of mRNAs. A cluster analysis of the ceRNA network was then performed using the molecular complex detection algorithm (MCODE) plug of Cytoscape software. This algorithm could detect densely connected regions in complex interaction networks.^33^

### 2.7 Gene function annotation

The ‘*EnrichGO*’ (GO, gene ontology) and ‘*enrichKEGG*’ (KEGG, Kyoto Encyclopedia of Genes and Genomes) functions of the Bioconductor ‘clusterProfiler’ package of R 3.2.2 software were used for the function annotation and pathway analysis of DEmRNAs and for exploring the roles of RNAs in the pathogenesis of SSH.^34^ *P* < .05 was considered to demonstrate significant gene enrichment results.

### 2.8 Quantitative real‐time polymerase chain reaction (qRT‐PCR)

The difference and regulatory relationship among hub lncRNAs, mRNAs and miRNAs from the ceRNA network were validated by qRT‐PCR. Whole blood samples from 51 SSH patients and 61 SRH patients were collected for the extraction of total RNAs for qRT‐PCR using the SYBR Green method. The expression levels of hsa‐miR‐361‐5p, which was the most significant miRNA identified in the previous validation study, and the hub lncRNAs and mRNAs were simultaneously investigated in 20 SSH patients and 19 SRH patients to explore the RNA interactions. Glyceraldehyde‐3‐phosphate dehydrogenase (GAPDH) was used as a stable internal control. The details of the experimental process are provided in the instructions. For each condition, three replicate experiments were conducted, and the mean cycle threshold (*C*_t_) was calculated. The 2^−Δ^ *^C^* ^t^ method was used for the calculation of relative quantitative expression (Δ*C*_t_ = Ct_RNA_ − Ct_GAPDH_).

### 2.9 Statistical analyses

The sample size used for qRT‐PCR validation was calculated using two independent *t* test formulas. SPSS 24.0 was used for the statistical analyses and hypothesis testing. The original microarray data were normalized using the ‘limma’ package of R software. If the data were normally distributed, two independent *t* tests were used to identify the differentially expressed RNAs between the SSH and SRH groups. The non‐normally distributed data were normalized. The data that could not be normalized were analysed using a Wilcoxon rank‐sum test. The qualitative variables were compared using Pearson *chi‐squared* or Fisher's exact tests. Non‐conditional logistic regression was applied to discover the factors that affect the risk of salt‐sensitive hypertension. The regulatory relationship between DERNAs was analysed through linear and partial correlation analyses. The area under the curve (AUC) of the receptor operation characteristics curve (ROC) was used to evaluate the diagnostic effects of differentially expressed RNAs. Cytoscape 3.4.0 software was used to visualize the coregulatory network, and heatmaps of DERNAs were drawn using Cluster 3.0 and Java TreeView.

## 3 RESULTS

### 3.1 Identification of differentially expressed lncRNA and mRNA profiles in SSH and SRH patients by microarray

**Figure 1:**
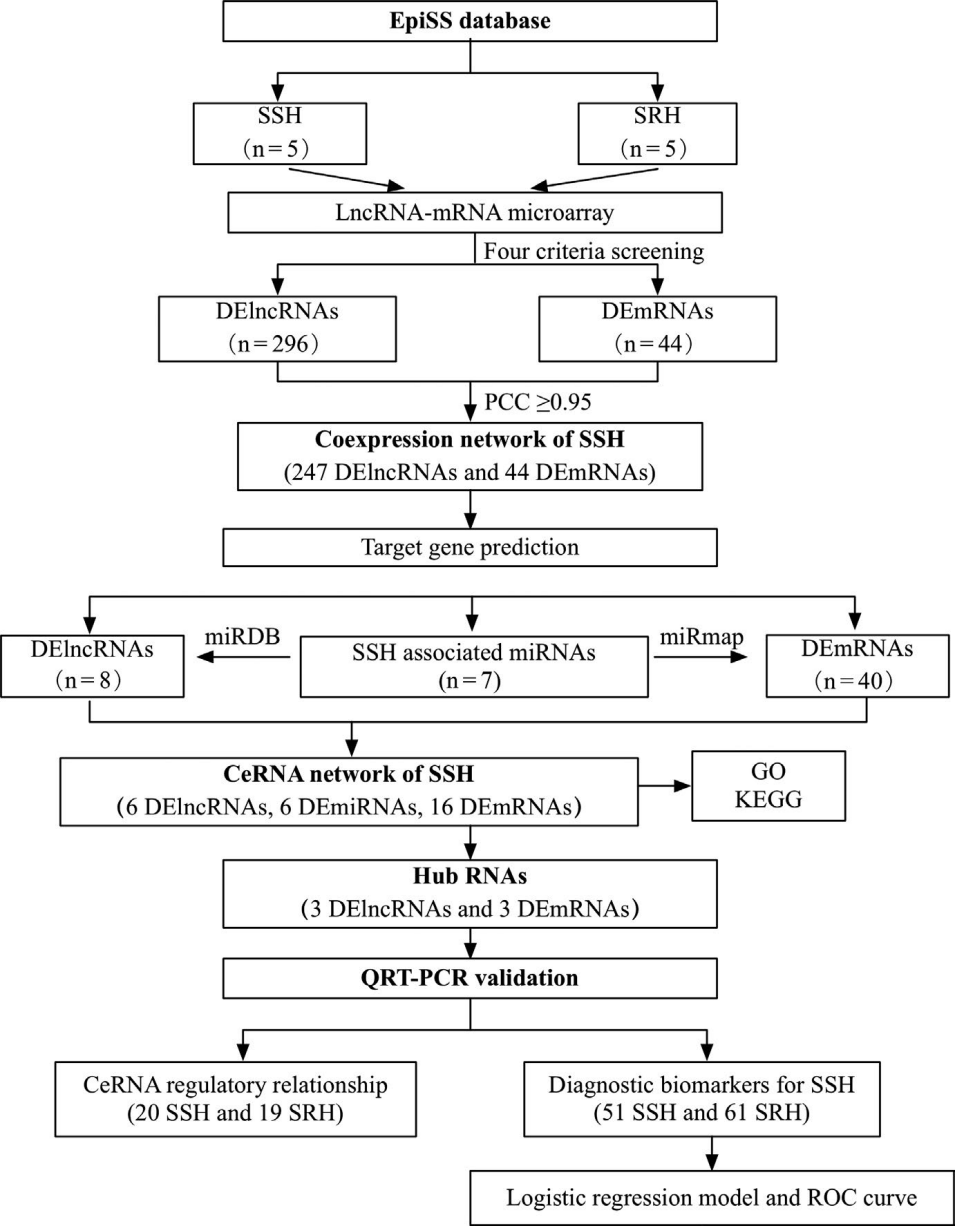
Flowchart of the study design. CeRNA, competing endogenous RNA; DElncRNAs, differentially expressed lncRNAs; DEmRNAs, differentially expressed mRNAs; EpiSS, System Epidemiology Study on Salt Sensitivity of Blood pressure; GO, gene ontology; KEGG, Kyoto Encyclopedia of Genes and Genomes; PCC, Pearson correlation coefficient; qRT‐PCR, quantitative real‐time polymerase chain reaction; ROC, receiver operating characteristic curve; SSH, salt‐sensitive hypertension; SRH, salt‐resistant hypertension

**Figure 2:**
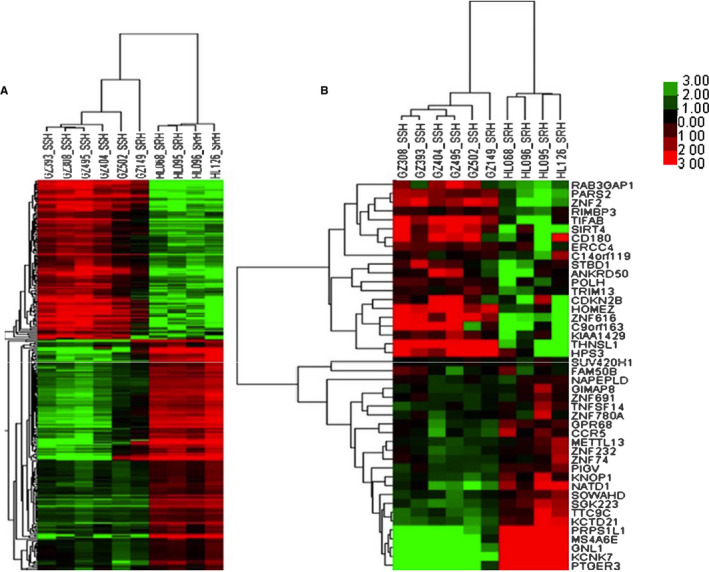
Heatmaps of 296 DElncRNAs (A) and 44 DEmRNAs (B) from microarray data between salt‐sensitive hypertension (N = 5) and salt‐resistant hypertension (N = 5). The columns represent samples, and the rows represent differentially expressed transcripts. The names of the DElncRNAs are not listed due to space constraints. The red and green colours indicate up‐regulated and down‐regulated RNAs, respectively, between the two groups

Whole blood samples from five SSH patients and five SRH patients were collected for lncRNA and mRNA microarray analyses. These patients were six females and four males with an average age of 63.60 ± 1.58 years. The microarray chip contained 68 423 lncRNAs and 18 853 mRNAs. Figure 1 showed a flow chart of the study design. The analysis identified 296 lncRNAs and 44 mRNAs that were differentially expressed between the SSH and SRH groups, and these were regarded as DElncRNAs and DEmRNAs, respectively. As shown in the cluster heatmaps (Figure 2), 121 and 175 DElncRNAs were up‐regulated and down‐regulated, respectively, and 39 and five DEmRNAs were up‐regulated and down‐regulated, respectively, which suggested that lncRNAs and mRNAs were dysregulated between SSH and SRH.

### 3.2 Coexpression between DElncRNAs and DEmRNAs

**Figure 3:**
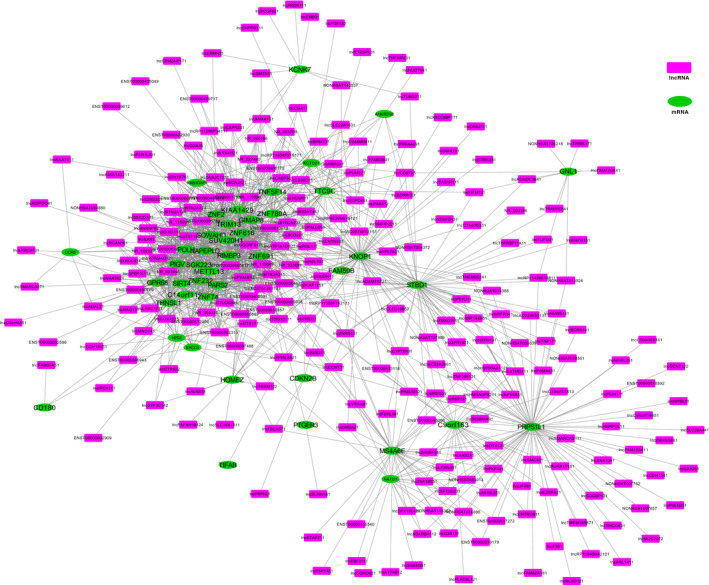
Coexpression network of 247 DElncRNAs and 44 DEmRNAs of salt‐sensitive hypertension with a PCC (Pearson correlation coefficient) ≥0.95. The green ovals represent DEmRNAs, the purple rectangles represent DElncRNAs, and the edges represent coexpression relationships between lncRNAs and mRNAs

The potential interactions between DElncRNAs and DEmRNAs were investigated through Pearson linear correlation analysis. In this study, 247 DElncRNAs and 44 DEmRNAs with a PCC ≥ 0.95 were included in the coexpression network **(**Figure 3 **)**. As shown in Figure 3, one lncRNA or mRNA might correlate with one or dozens of RNAs, satisfying the scale‐free condition. The left part of the coexpression network showed a dense distribution, which could have important biological functions and be the selection range of hub genes. Noticeably, only up‐regulated DElncRNAs were selected for further ceRNA analysis due to their more biological functions. Fifty up‐regulated DElncRNAs could be matched with sequence information in the LNCipedia database; therefore, these DElncRNAs could be used to predict target miRNAs.

### 3.3 Prediction of target lncRNAs and mRNAs of DEmiRNAs

A previous study discovered eight SSH‐associated DEmiRNAs,^15^ and we used these DEmiRNAs to find the target DElncRNAs that had prediction relationships with these miRNAs. This analysis identified eight target DElncRNAs (lnc‐ILK‐8:1, lnc‐ACP1‐1:1, lnc‐OTX1‐7:1, lnc‐SLC35A3‐1:2, lnc‐MREG‐3:1, lnc‐DCAF17‐5:1, lnc‐ATP48‐1:2 and lnc‐RCAN1‐6:1) among the DElncRNAs (Table S1).

Similarly, previously discovered DEmiRNAs were regarded as the cores to predict their target mRNAs using the miRmap database (Table S2). Prediction results were outputted and converged with 44 DEmRNAs to obtain the target DEmRNAs. Ultimately, seven DEmiRNAs had predicted relationships with 40 target DEmRNAs.

### 3.4 Construction of the ceRNA network and identification of hub RNAs for SSH

**Figure 4:**
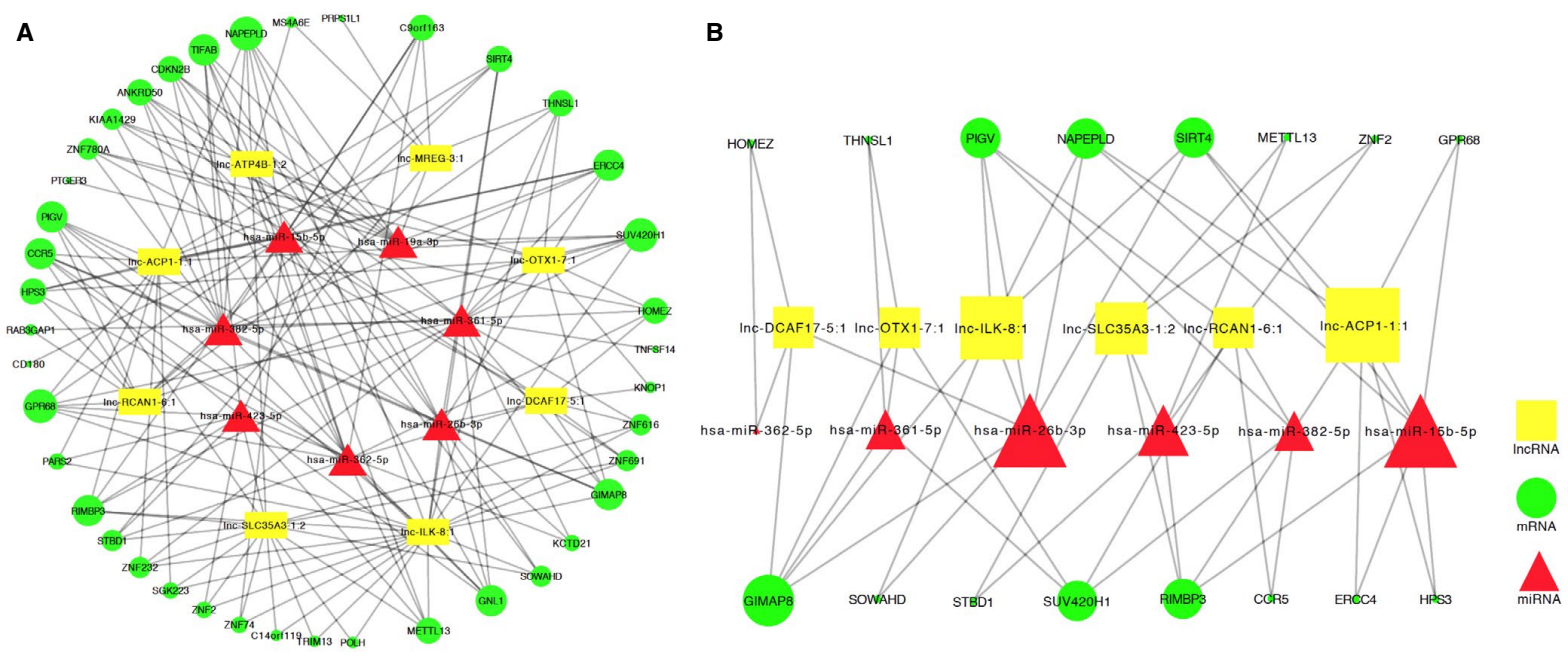
ceRNA coregulatory network of salt‐sensitive hypertension. The red triangles represent miRNAs, the green circles represent mRNAs, and the yellow squares represent lncRNAs. The size of a node represents the number of interactions. A, Global view of the ceRNA network of salt‐sensitive hypertension. B, ceRNA network of salt‐sensitive hypertension after removing the nodes and lines that could not form ceRNA loops

After removing the overlapping lncRNAs, we inputted eight DElncRNAs, seven DEmiRNAs and 40 DEmRNAs into Cytoscape 3.4.0 software to construct the coregulatory network (Figure 4). The size of nodes in the network represents the number of interactions: the green rounds represent mRNAs, the yellow rectangles are lncRNAs, and red triangles designate miRNAs. We then removed the nodes that could not form three‐dimensional regulatory relationships and finally obtained the SSH ceRNA network (Figure 4B). The network contained six DElncRNAs (lnc‐ILK‐8:1, lnc‐ACP1‐1:1, lnc‐OTX1‐7:1, lnc‐SLC35A3‐1:2, lnc‐DCAF17‐5:1 and lnc‐RCAN1‐6:1), six DEmiRNAs (miR‐26b‐3p, miR‐15b‐5p, miR‐361‐5p, miR‐423‐5p, miR‐362‐5p and miR‐382‐5p) and 16 DEmRNAs. The detailed regulatory relationships were described in Table 1. MCODE identified one cluster in the ceRNA network, which included six nodes and eight links. According to the expression levels, *P* values, fold changes and MCODE results, the following three DElncRNAs and three DEmRNAs were selected as hub RNAs for validation: lnc‐ILK‐8:1, lnc‐OTX1‐7:1, lnc‐RCAN1‐6:1, *GIMAP8*, *SUV420H1* and *PIGV*.

**Table 1:** ceRNA regulatory relationships among lncRNAs, miRNAs and mRNAs in salt‐sensitive hypertension

lncRNAs	miRNAs	mRNAs
lnc‐ILK‐8:1	hsa‐miR‐26b‐3p	*PIGV*, *NAPEPLD*, *SIRT4*, *GIMAP8*, *SOWAHD*
lnc‐ACP1‐1:1	hsa‐miR‐15b‐5p	*NAPEPLD*, *SIRT4*, *GPR68, RIMBP3*, *ERCC4*, *HPS3*
lnc‐OTX1‐7:1	hsa‐miR‐361‐5p	*THNSL1*, *GIMAP8*, *SUV420H1*
lnc‐SLC35A3‐1:2	hsa‐miR‐423‐5p	*METTL13*, *ZNF2*, *STBD1*, *RIMBP3*
lnc‐DCAF17‐5:1	hsa‐miR‐362‐5p hsa‐miR‐26b‐3p	*HOMEZ*, *GIMAP8*
lnc‐RCAN1‐6:1	hsa‐miR‐382‐5p	*PIGV*, *CCR5*, *SUV420H1*

### 3.5 GO enrichment and KEGG pathway analyses of DEmRNAs and target mRNAs of DEmiRNAs

Before validation, we integrated the DEmRNAs and target mRNAs of DEmiRNAs to investigate the potential functions of hub RNAs in the pathogenesis of SSH through KEGG pathway and GO enrichment analyses. The KEGG pathway analyses showed that the hub genes were involved in the cancer pathway, cAMP signalling pathway, mTOR signalling pathway, VEGF signalling pathway, aldosterone‐regulated sodium reabsorption, WNT signalling pathway, Ras signalling pathway and HIF‐1 signalling pathway. These results also indicated that the hub genes in the ceRNA network were enriched in 348 GO functional terms. The five most significant terms of each GO category were listed in Table S3, and these were primarily involved in basic biological processes.

### 3.6 Validation of ceRNA regulatory relationships of SSH by qRT‐PCR

**Figure 5:**
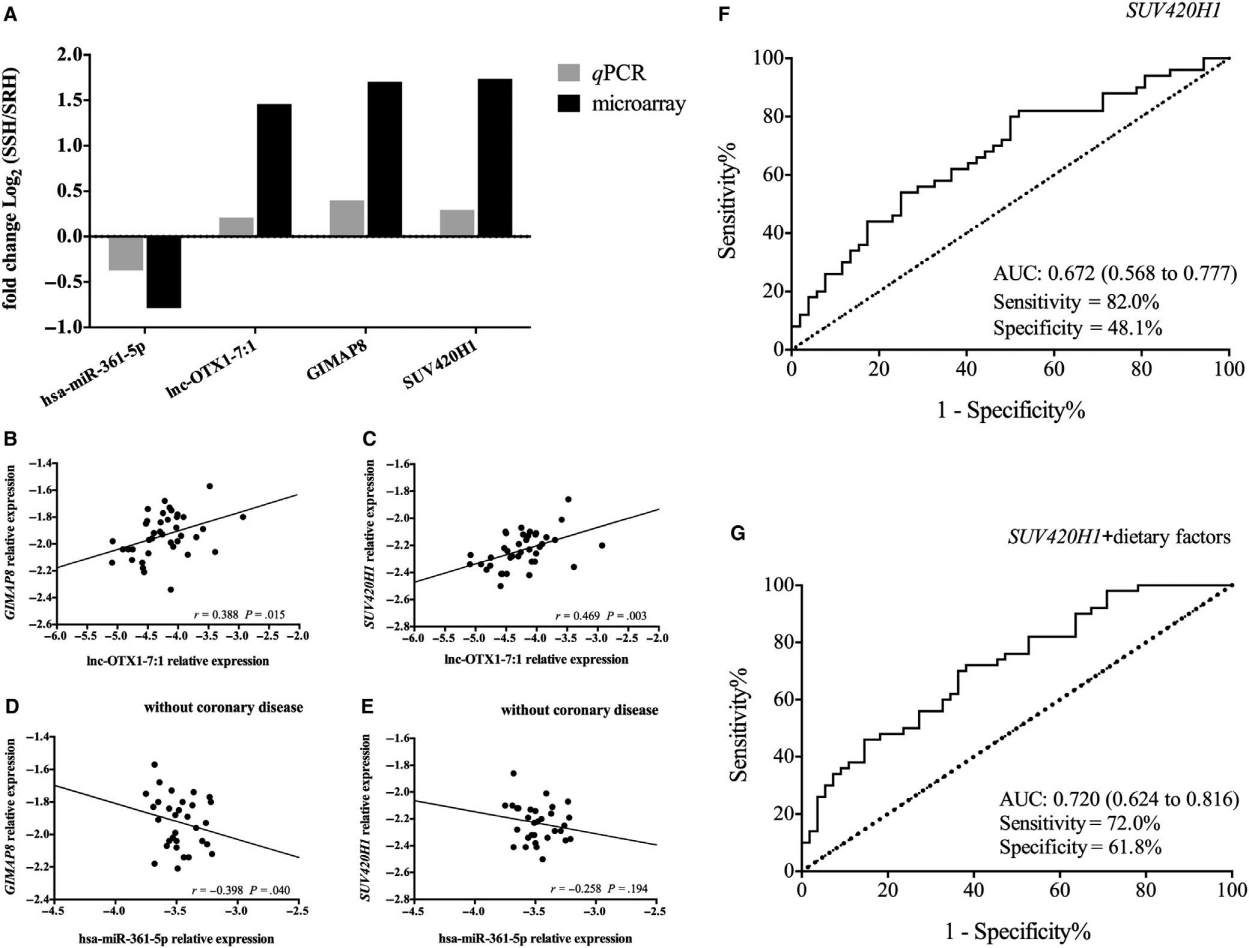
Validation of ceRNA regulatory relationships for lnc‐OTX1‐7:1, *SUV420H1*, *GIMAP8* and hsa‐miR‐361‐5p and diagnostic performance of *SUV420H1* for salt‐sensitive hypertension. A, Fold changes of four hub RNAs obtained through qRT‐PCR validation and microarray analyses. B‐E, Linear correlations and partial correlations among four RNAs. F‐G, Receptor operating characteristic curve of the diagnostic performance of *SUV420H1* with or without dietary factors

Four (Lnc‐OTX1‐7:1, hsa‐miR‐361‐5p, *GIMAP8* and *SUV420H1*) of the six hub RNAs were selected to validate the ceRNA crosstalk relationships by qRT‐PCR. According to the ceRNA network, these four hub RNAs could form two ceRNA loops: ‘lnc‐OTX1‐7:1 → hsa‐miR‐361‐5p → *GIMAP8*’ and ‘lnc‐OTX1‐7:1 → hsa‐miR‐361‐5p → *SUV420H1*’. The validation results obtained with 20 SSH patients and 19 SRH patients revealed that hsa‐miR‐361‐5p (*t* = 2.128, *P* = .040), *GIMAP8* (*t* = −2.448, *P* = .019) and *SUV420H1* (*t* = −2.051, *P* = .049) were differentially expressed between the SSH and SRH groups. However, we could not detect a statistical correlation between lnc‐OTX1‐7:1 and hsa‐miR‐361‐5p, possibly because different concentrations of lncRNAs and miRNAs, as well as their interactions with RNAs, affect the activity of ceRNA functions.^35^ In addition, hsa‐miR‐361‐5p was down‐regulated in SSH, whereas lnc‐OTX1‐7:1, *GIMAP8* and *SUV420H1* were up‐regulated in SSH (Figure 5A), which was consistent with the miRNA‐mediating crosstalk between lncRNAs and mRNAs. The linear and partial correlations showed that lnc‐OTX1‐7:1 was significantly positively associated with *GIMAP8* and *SUV420H1* (*r* = .388, *P* = .015; *r* = .469, *P* = .003). In the patients without coronary disease, hsa‐miR‐361‐5p was significantly negatively associated with *GIMAP8* and *SUV420H1* (*r* = −.398, *P* = .040 and *r* = −.258, *P* = .194; Figure 5B‐E).

Multiple linear regression indicated that independent variables, including the expression of lnc‐OTX1‐7:1 and salt sensitivity, were significantly associated with the expression of *GIMAP8* (dependent variable) with a *B* coefficient (95% CI) of 0.127 (0.022‐0.232) and *P* = .020 and a *B* coefficient (95% CI) of 0.026 (0.014‐0.211) and *P* = .026, respectively. Similarly, lnc‐OTX1‐7:1 was significantly associated with the expression level of *SUV420H1* with a *B* coefficient (95% CI) of 0.130 (0.045‐0.216) and *P* = .004. The correlation and regression results suggested the existence of a positive association between lncRNAs and mRNAs.

### 3.7 Baseline characteristics of the validation population and differences in hub lncRNAs and mRNAs between SSH and SRH

The baseline characteristics of 51 SSH and 61 SRH patients are described in Table 2. The results from two independent sample *t* tests and *chi‐squared* tests showed that gender, age, BMI, blood pressure, dietary factors and history of cardiovascular disease did not significantly differ between the SSH and SRH groups (*P* > .05). Seven participants were removed because their RNA samples were not analysed by qRT‐PCR, and the total sample size was 105 (50 SSH patients and 55 SRH patients). Two independent sample *t* tests and Wilcoxon rank‐sum tests were used to investigate the difference in the six hub RNAs between the SSH and SRH groups. Finally, we found that three mRNAs (*PIGV*, *SUV420H1* and *GIMAP8*) were significantly differentially expressed between the two groups, whereas three lncRNAs exhibited no significant differences (Table S4).

**Table 2:** Baseline characteristics of subjects for RNA validation

Variables	Total	SRH	SSH	*P* values
Sample size (%)	112 (100.0)	60 (53.6)	52 (46.4)	—
Gender, male (%)	31 (27.7)	17 (54.8)	14 (45.2)	.961^†^
Age (years)	63.13 ± 5.60	63.46 ± 5.52	62.75 ± 5.72	.504*
BMI (kg/m^2^)	26.52 ± 3.32	26.31 ± 3.41	26.76 ± 3.22	.475*
Baseline SBP	139.36 ± 16.75	138.97 ± 15.33	139.82 ± 18.45	.792*
Baseline DBP	80.49 ± 10.69	79.39 ± 9.91	81.81 ± 11.52	.235*
Follow‐up SBP	143.66 ± 17.94	144.25 ± 18.51	142.96 ± 17.40	.708*
Follow‐up DBP	78.51 ± 10.64	76.99 ± 9.19	80.33 ± 11.99	.098*
Frequent of poultry, No. (%)				
Every day	9 (8.0)	4 (6.7)	5 (9.6)	.127^†^
4‐6 times/week	6 (5.4)	3 (5.0)	3 (5.8)	
1‐3 times/week	55 (49.1)	25 (41.7)	30 (57.7)	
1‐3 times/month	33 (29.5)	20 (33.3)	13 (25.0)	
Never	9 (8.0)	8 (13.3)	1 (1.9)	
Diabetes, No. (%)				
Yes	32 (28.6)	17 (27.9)	15 (29.4)	.857^†^
No	80 (71.4)	44 (72.1)	36 (70.6)	
Coronary disease, No. (%)				
Yes	24 (21.4)	15 (24.6)	9 (17.6)	.373^†^
No	88 (78.6)	46 (75.4)	42 (82.4)	
Stroke, No. (%)				
Yes	19 (17.0)	8 (13.1)	11 (21.6)	.235^†^
No	93 (83.0)	53 (86.9)	40 (78.4)	

Abbreviations: BMI, body mass index; SRH, salt‐sensitive hypertension; SSH, salt‐resistant hypertension.

* Two independent sample t tests.

^†^ Chi‐square tests, and *P* < .05 were considered to indicate statistical significance.

### 3.8 Establishing a diagnostic hub RNA model of SSH through logistic regression analysis

The associations of the six hub RNAs (lnc‐ILK‐8:1, lnc‐OTX1‐7:1, lnc‐RCAN1‐6:1, *GIMAP8*, *SUV420H1* and *PIGV*) with SSH were analysed using a logistic regression model adjusting for dietary factors and history of coronary heart disease, which were found to be associated with SSH, although the differences of these two variables did not reach the statistically significant level in our study. First, we entered all six hub RNAs and adjusted factors into the model and found that *SUV420H1* was a dangerous risk factor for SSH (OR, 95% CI: 6.637, 1.259‐34.983; *P* = .026), and a similar finding was obtained for the frequency of having poultry (1.979, 1.175‐3.331; *P* = .010). A stepwise method was then used to construct the diagnostic model of SSH, which included *SHV420H1* and dietary factors: logit (*P* = SSH) = 7.996 + 1.307 × *SUV420H1* + 0.642 × poultry.

### 3.9 ROC analysis evaluating the diagnostic performance of the hub RNA model for SSH

The predicted probabilities of the stepwise logistic model were applied to draw the ROC curve. The AUC of *SUV420H1* was 0.672 (95% CI, 0.568‐0.777; sensitivity = 82%; specificity = 48.1%). However, the inclusion of dietary factors in the model increased the AUC to 0.720 (95% CI, 0.624‐0.816; sensitivity = 72%; specificity = 61.8%) (Figure 5F,G). The diagnostic performance of *SUV420H1* alone was slightly better than that of hsa‐miR‐361‐5p (AUC: 0.672 > 0.617), as reported in a previously published article.^15^

## 4 DISCUSSION

In the present study, we aimed to comprehensively integrate lncRNA‐mRNA microarray data with previous miRNA sequencing results to build a ceRNA coregulatory network and discover hub lncRNAs and mRNAs for the diagnosis of salt‐sensitive hypertension based on ceRNA theory, which can provide insights into the mechanism of SSH. Finally, the analysis of the ceRNA network identified 23 SSH‐associated ceRNA loops and six hub RNAs, including three lncRNAs (lnc‐ILK‐8:1, lnc‐OTX1‐7:1 and lnc‐RCAN1‐6:1) and three mRNAs (*GIMAP8*, *SUV420H1* and *PIGV*). The six RNAs were not only differentially expressed between the SSH and SRH groups but also exhibited correlation and prediction relationships with miR‐361‐5p, miR‐26b‐3p and miR‐382‐5p, which were previously discovered to be potential biomarkers for the diagnosis of SSH. Thus, the six hub RNAs are strongly important for the diagnosis and targeted therapy of SSH.

In addition, among the six hub RNAs, lnc‐OTX1‐7:1 could potentially act as a sponge of hsa‐miR‐361‐5p and down‐regulate the expression of *GIMAP8* and *SUV420H1*. This result was validated in 39 hypertensive patients by qRT‐PCR. The fold changes and correlation results all indicated that the interactions of the four RNAs corresponded to the ceRNA regulatory theory. The ceRNA network consisting of lncRNAs, miRNAs and mRNAs has been widely used to investigate the mechanism of cardiovascular diseases, such as cardiac hypertrophy,^36^ pulmonary arterial hypertension^37^ and ischaemic stroke.^38^ For example, the lncRNA CYTOR could modulate pathological cardiac hypertrophy by serving as a ceRNA for miR‐155.^39^ In addition, the alteration of lncRNA MALAT1 could up‐regulate the expression of X box‐binding protein by functioning as a ceRNA for miR‐124, which contributes to pulmonary arterial hypertension.^40^ However, the ceRNAs in SSH were poorly understood, which might be due to the difficulties associated with the definition of SSH. Rapid and chronic oral normal saline loading and depletion are commonly used to diagnose SSH, but all of these tests involve complex procedures and are associated with poor participant compliance. Our study investigated the transcriptome profiles of SSH based on a previously established EpiSS database and therefore fills in the blanks and provides a hypothesis for further validation of the mechanism of SSH.

The larger‐sample validation revealed that three mRNAs were differentially expressed between the SSH and SRH groups (*PIGV*, *GIIMAP8* and *SUV420H1*), and after adjusting for dietary factors, *SUV420H1* was the only RNA included in the diagnostic model of SSH. Furthermore, *SUV420H1* is the target gene of three SSH‐associated miRNAs (hsa‐miR‐361‐5p, hsa‐miR‐382‐5p and hsa‐miR‐19a‐3p) and is strongly correlated with 51 DElncRNAs in the SSH coexpression network. This gene is also involved in two ceRNA loops in SSH, and one of these loops has been verified in SSH patients. This evidence suggests that *SUV420H1* might be a core element regulating the pathogenesis mechanism of SSH.

*SUV420H1*, also known as *KMT5B* in humans, can encode the protein named histone H4 lysine 20 trimethyl transferase (Suv4‐20h1). Chinenov et al found that this protein can affect glucocorticoid receptor (GR) target gene expression by participating in the transcriptional regulation of GR.^41^ Evidently, glucocorticoid is an important regulator in renal Na^+^ transport^42^ that can improve Na^+^ reabsorption and retention, which is stimulated by 11β‐hydroxysteroid dehydrogenase (11*β*HSD). The positive regulatory relationships among 11*β*HSD, glucocorticoid and SSH have been widely elucidated in rats^43^ and humans.^44^, ^45^ Thus, we hypothesize that *SUV420H1* plays a role in the pathogenesis of SSH, probably by participating in the GR pathway, increasing Na^+^ reabsorption and ultimately resulting in SSH. Additionally, hsa‐miR‐361‐5p was found to be associated with insulin sensitivity through involvement in the WNT signalling pathway,^15^ which might also be a potential pathway for hsa‐miR‐361‐5p in SSH.^46^ However, direct evidence on the relationships among hsa‐miR‐361‐5p, lnc‐OTX1‐7:1 and *SUV420H* is lacking. We will explore the ceRNA regulatory relationships and their interactions on GR through cellular experiments to further illuminate the mechanism of SSH.

Similarly, GO enrichment and KEGG pathway analyses showed that the DEmRNAs of SSH are associated with aldosterone‐regulated sodium reabsorption, which is an important mechanism underlying the pathogenesis of SSH.^47^ Aldosterone is a steroid hormone that is produced by the zona glomerulosa of the adrenal cortex, which is responsible for homeostatic regulation. The hypersecretion of aldosterone will result in the elevation of blood pressure.^48^ The renal function curve of SSH patients moves to the right, which demonstrates that increases in blood pressure result in decreases in sodium excretion.^49^ The enrichment analysis illustrated that the hub biomarkers screened from the ceRNA network might result in glomerulosclerosis and renal damage by regulating the level of aldosterone and affecting renal blood flow. Additionally, a lncRNA‐mRNA coexpression analysis revealed that the phosphatidylinositol‐3‐kinase (PI3K)‐Akt signalling pathway exhibits the strongest association with salt sensitivity.^50^ Aldosterone can regulate sodium transport by stimulating PI3K and ultimately active epithelial sodium channels (ENaCs).^51^ Considering the close associations between aldosterone and RNA biomarkers, further functional experiments could be performed to validate the regulatory relationship among hub biomarkers and their downstream proteins in the RAAS.

Interestingly, the logistic model revealed that the frequency of poultry was positively associated with the risk of SSH (OR = 1.979, 95% CI = (1.175, 3.331), *P* = .010). The influence of poultry on SSH might be attributed to the habit of cooking with high‐salt levels. In China, particularly the northern part, people prefer smoked and salt‐roasted, as well as stewed, chickens with soy sauce, which could increase invisible salt intake ^52^ and eventually result in an increase in blood pressure.^53^ Thus, to better control their blood pressure, the population in northern China could change their dietary habits by reducing the use of paste and sauce, eating home‐cooked meals rather than eating at restaurants,^54^ using low‐sodium salt substitutes ^55^ and potassium supplementation.^56^

This study has some limitations. First, due to the difficulty of salt‐sensitive determination, we validated the interactions among lncRNAs, miRNAs and mRNAs in 39 participants. Further larger‐sample validation is needed to verify the ceRNA relationships. Second, due to the case‐control study design, we could only observe the associations between hub RNAs and SSH. The causality needs to be further explored through loss‐of‐function and‐gain‐of‐function experiments and animal models. Finally, considering the relatively high risks of SSH and the worse prognosis of cardiovascular events in the middle‐ to older‐aged populations, we included essential hypertensive patients aged 40‐70 years for the RNA microarray experiment to improve the efficiency of diagnosis and the effectiveness of treatment; thus, the results are only suitable for this population. In future research, we will collect samples from different age stages to explore the differences in ceRNA mechanisms among populations.

In conclusion, six hub DElncRNAs and DEmRNAs were identified from 23 ceRNA loops associated with SSH. The interactions of the six RNAs and hsa‐miR‐361‐5p were validated and provide a hypothesis for the pathogenesis of SSH. In particular, *SUV420H1* was not only involved in the ceRNA network but also included in the diagnostic panel of SSH, which indicates that this ceRNA might be a hub element that participates in the pathogenesis of SSH and an important biomarker for the early recognition of SSH.

## CONFLICT OF INTEREST

The authors confirm that there are no conflicts of interest.

## AUTHOR CONTRIBUTIONS

Ling Zhang conceived and designed the study. Han Qi, Zheng Liu, Wen‐Juan Peng, Han Cao, Chun‐Yue Guo and Yan‐Yan Sun involved in the field survey and acquisition of data. Han Qi performed the data analyses and interpretation. Ling Zhang and Han Qi drafted the manuscript. Christine Pao and Yu‐Tao Xiang revised the language. All authors read and approve final manuscript.

## Supporting information

Table S1‐S4
