# Supplementary material for: Construction of a ceRNA coregulatory network and screening of hub biomarkers for salt‐sensitive hypertension
Source: J Cell Mol Med. 2020 May 15;24(13):7254–65. doi: 10.1111/jcmm.15285 (PMC7379024; doi:10.1111/jcmm.15285)
Supplement: Supplementary file 1 — Table S1‐S4 [file JCMM-24-7254-s001.docx]

Supplementary Table S1. The target prediction results of lncRNAs to miRNAs

| **LncRNA** | **Target miRNA** |
| --- | --- |
| lnc-ILK-8:1 | hsa-miR-26b-3p |
| lnc-ACP1-1:1 | hsa-miR-15b-5p |
| lnc-OTX1-7:1 | hsa-miR-361-5p |
| lnc-SLC35A3-1:2 | hsa-miR-423-5p |
| lnc-MREG-3:1 | hsa-miR-19a-3p |
| lnc-DCAF17-5:1 | hsa-miR-26b-3p, hsa-miR-362-5p |
| lnc-ATP48-1:2 | hsa-miR-362-5p |
| lnc-RCAN1-6:1 | hsa-miR-382-5p |

Supplementary Table S2. The target prediction results of miRNAs to mRNAs

| **miRNA** | **mRNA** | **miRNA** | **mRNA** | **miRNA** | **mRNA** |
| --- | --- | --- | --- | --- | --- |
| hsa-miR-15b-5p | *METTL13* | hsa-miR-382-5p | *PIGV* | hsa-miR-26b-3p | *SIRT4* |
| hsa-miR-15b-5p | *NAPEPLD* | hsa-miR-382-5p | *NAPEPLD* | hsa-miR-26b-3p | *GNL1* |
| hsa-miR-15b-5p | *ZNF691* | hsa-miR-382-5p | *ZNF780A* | hsa-miR-26b-3p | *SOWAHD* |
| hsa-miR-15b-5p | *GPR68* | hsa-miR-382-5p | *KIAA1429* | hsa-miR-26b-3p | *CCR5* |
| hsa-miR-15b-5p | *ZNF74* | hsa-miR-382-5p | *GNL1* | hsa-miR-26b-3p | *KCTD21* |
| hsa-miR-15b-5p | *RIMBP3* | hsa-miR-382-5p | *CCR5* | hsa-miR-26b-3p | *GIMAP8* |
| hsa-miR-15b-5p | *SIRT4* | hsa-miR-382-5p | *ANKRD50* | hsa-miR-26b-3p | *ERCC4* |
| hsa-miR-15b-5p | *GNL1* | hsa-miR-382-5p | *GIMAP8* | hsa-miR-26b-3p | *CDKN2B* |
| hsa-miR-15b-5p | *SOWAHD* | hsa-miR-382-5p | *ERCC4* | hsa-miR-26b-3p | *TIFAB* |
| hsa-miR-15b-5p | *ANKRD50* | hsa-miR-382-5p | *CDKN2B* | hsa-miR-19a-3p | *SUV420H1* |
| hsa-miR-15b-5p | *HPS3* | hsa-miR-382-5p | *HOMEZ* | hsa-miR-19a-3p | *NAPEPLD* |
| hsa-miR-15b-5p | *CD180* | hsa-miR-382-5p | *TIFAB* | hsa-miR-19a-3p | *ZNF780A* |
| hsa-miR-15b-5p | *ERCC4* | hsa-miR-382-5p | *C9orf163* | hsa-miR-19a-3p | *KIAA1429* |
| hsa-miR-15b-5p | *HOMEZ* | hsa-miR-361-5p | *SUV420H1* | hsa-miR-19a-3p | *ANKRD50* |
| hsa-miR-15b-5p | *TIFAB* | hsa-miR-361-5p | *ZNF616* | hsa-miR-19a-3p | *ERCC4* |
| hsa-miR-15b-5p | *C9orf163* | hsa-miR-361-5p | *GPR68* | hsa-miR-19a-3p | *CDKN2B* |
| hsa-miR-15b-5p | *PTGER3* | hsa-miR-361-5p | *RAB3GAP1* | hsa-miR-19a-3p | *TIFAB* |
| hsa-miR-423-5p | *STBD1* | hsa-miR-361-5p | *RIMBP3* | hsa-miR-362-5p | *STBD1* |
| hsa-miR-423-5p | *METTL13* | hsa-miR-361-5p | *ZNF232* | hsa-miR-362-5p | *METTL13* |
| hsa-miR-423-5p | *GPR68* | hsa-miR-361-5p | *GNL1* | hsa-miR-362-5p | *GPR68* |
| hsa-miR-423-5p | *RIMBP3* | hsa-miR-361-5p | *TNFSF14* | hsa-miR-362-5p | *GNL1* |
| hsa-miR-423-5p | *ZNF2* | hsa-miR-361-5p | *ANKRD50* | hsa-miR-362-5p | *CCR5* |
| hsa-miR-423-5p | *PARS2* | hsa-miR-361-5p | *KCTD21* | hsa-miR-362-5p | *ANKRD50* |
| hsa-miR-423-5p | *GNL1* | hsa-miR-361-5p | *GIMAP8* | hsa-miR-362-5p | *HPS3* |
| hsa-miR-423-5p | *CCR5* | hsa-miR-361-5p | *ERCC4* | hsa-miR-362-5p | *CDKN2B* |
| hsa-miR-423-5p | *KCTD21* | hsa-miR-361-5p | *CDKN2B* | hsa-miR-362-5p | *HOMEZ* |
| hsa-miR-423-5p | *TIFAB* | hsa-miR-361-5p | *THNSL1* | hsa-miR-362-5p | *TIFAB* |
| hsa-miR-423-5p | *C9orf163* | hsa-miR-26b-3p | *PIGV* | hsa-miR-362-5p | *C9orf163* |
| hsa-miR-382-5p | *SUV420H1* | hsa-miR-26b-3p | *NAPEPLD* | - | - |

Supplementary Table S3. The top five most significant terms of each GO categories.

| **categories** | **GO terms** | **number of genes** | ***P* value** |  |
| --- | --- | --- | --- | --- |
| BP | Vascular endothelial growth factor receptor signaling pathway | 8 | 5.00E-04 | |
|  | Dopaminergic neuron differentiation | 5 | 7.80E-04 | |
|  | Embryonic hindlimb morphogenesis | 5 | 2.00E-03 | |
|  | Startle response | 4 | 2.80E-03 | |
|  | Positive regulation of translational initiation | 4 | 2.80E-03 | |
| CC | Melanosome | 9 | 5.70E-04 | |
|  | Cytoplasm | 123 | 1.50E-03 | |
|  | Nucleoplasm | 73 | 1.60E-03 | |
|  | Extracellular vesicle | 5 | 1.40E-02 | |
|  | Endosome | 10 | 2.50E-02 | |
| MF | DNA binding | 50 | 1.60E-03 | |
|  | Protein binding | 195 | 1.70E-03 | |
|  | Nucleic acid binding | 33 | 2.30E-03 | |
|  | Transcription factor binding | 14 | 3.30E-03 | |
|  | Metal ion binding | 57 | 4.20E-03 | |

Note: GO, Gene ontology; BP, biological process; CC, cellular component; MF, molecular function.

Supplementary Table S4. The expression levels of 6 hub biomarkers for qRT-PCR validations.

| **Hub biomarkers** | **Total** | **SRH** | **SSH** | **Statistic** | ***P* value** |
| --- | --- | --- | --- | --- | --- |
| lnc-OTX1-7:1 | -12.61 ± 5.95 | -12.95 ± 5.98 | -12.25 ± 5.97 | -0.816^b^ | 0.414^#^ |
| lnc-RCAN1-6:1 | -8.37 ± 1.42 | -8.49 ± 1.28 | -8.24 ± 1.56 | -0.912^a^ | 0.364^*^ |
| lnc-ILK-8:1 | -10.16 ± 1.39 | -10.36 ± 1.24 | -9.94 ± 1.51 | -1.582^a^ | 0.117^*^ |
| *PIGV* | -9.15 ± 0.87 | -9.32± 0.73 | -8.97 ± 0.98 | -2.114^a^ | 0.037^*^ |
| *SUV420H1* | -7.47 ± 0.58 | -7.64 ± 0.49 | -7.29 ± 0.62 | -3.228^a^ | 0.002^*^ |
| *GIMAP8* | -6.81 ± 1.02 | -7.02 ± 0.82 | -6.58 ± 1.17 | -2.230^a^ | 0.028^*^ |

Note: * Two independent sample t-test, † Sum-rank test, a *t*-value, b z value, *P*<0.05 was considered statistically significant. SRH= Salt-sensitive hypertensives; SSH= Salt-resistant hypertensives.
